# Autophagy-independent function of *Atg1* for apoptosis-induced compensatory proliferation

**DOI:** 10.1186/s12915-016-0293-y

**Published:** 2016-08-19

**Authors:** Mingli Li, Jillian L. Lindblad, Ernesto Perez, Andreas Bergmann, Yun Fan

**Affiliations:** 1 University of Birmingham, School of Biosciences, Edgbaston, Birmingham, B15 2TT UK; 2 Department of Molecular, Cell and Cancer Biology, University of Massachusetts Medical School, 364 Plantation Street, LRB419, Worcester, MA 01605 USA

**Keywords:** Apoptosis-induced proliferation, Atg1, ULK1/2, Autophagy, Jun-N-terminal kinase signaling

## Abstract

**Background:**

ATG1 belongs to the Uncoordinated-51-like kinase protein family. Members of this family are best characterized for roles in macroautophagy and neuronal development. Apoptosis-induced proliferation (AiP) is a caspase-directed and JNK-dependent process which is involved in tissue repair and regeneration after massive stress-induced apoptotic cell loss. Under certain conditions, AiP can cause tissue overgrowth with implications for cancer.

**Results:**

Here, we show that *Atg1* in *Drosophila* (*dAtg1*) has a previously unrecognized function for both regenerative and overgrowth-promoting AiP in eye and wing imaginal discs. *dAtg1* acts genetically downstream of and is transcriptionally induced by JNK activity, and it is required for JNK-dependent production of mitogens such as Wingless for AiP. Interestingly, this function of *dAtg1* in AiP is independent of its roles in autophagy and in neuronal development.

**Conclusion:**

In addition to a role of *dAtg1* in autophagy and neuronal development, we report a third function of *dAtg1* for AiP.

**Electronic supplementary material:**

The online version of this article (doi:10.1186/s12915-016-0293-y) contains supplementary material, which is available to authorized users.

## Background

*Autophagy-related gene 1* (*Atg1*) in yeast, *dAtg1* in *Drosophila*, *uncoordinated-51* (*unc-51*) in *C. elegans*, and Unc-51-like kinase 1 and 2 (ULK1/2) in mammals are members of the evolutionary conserved Uncoordinated-51-like kinase (ULK) protein kinase family that play critical roles in macroautophagy (referred to as autophagy) and neuronal development (reviewed in [1, 2]). Autophagy is a catabolic process engaged under starvation and other stress conditions [3]. A critical step in autophagy is the formation of autophagosomes which trap cytosolic cargo for degradation after fusion with lysosomes [3]. Genetic studies in yeast identified *Atg1* as an essential gene required for the initiation of autophagy [3–5]. This function of ULK proteins is conserved in evolution [6–9]. For this process, ATG1 forms a protein complex composed of ATG1/ULK1, ATG13, and ATG17 (FIP200), and in mammalian cells also ATG101 [10–15]. The ATG1/ULK complex phosphorylates several substrates including ATG9 [16, 17] and the Myosin light chain kinase (ZIP kinase in mammals, Sqa in *Drosophila*) [18], which are required for the formation of autophagosomes. Activation of the ATG1/ULK complex is also required for the recruitment of the ATG6/Beclin protein complex to the pre-autophagosomal structure (PAS) [3]. The ATG6/Beclin complex is composed of ATG6 (Beclin-1 in mammals), the type III PI3-K VPS34, as well as ATG14 and VPS15. Maturation of the PAS to autophagosomes requires lipidation of the ubiquitin-like ATG8/LC3 protein, which is mediated by two ubiquitin-like conjugation systems, ATG12 and ATG8/LC3 [3]. Critical components in these ubiquitin-like conjugation systems are ATG7 (E1), ATG10 and ATG3 (E2s), as well as another protein complex, ATG5-ATG12-ATG16, which serves as an E3 ligase for ATG8/LC3 lipidation [3, 19–21]. Finally, autophagosomes fuse with lysosomes for degradation of cargo.

In addition to a critical role in autophagy, ATG1 also has functions outside of autophagy, most notably in neuronal development. This was initially observed in mutants of the ULK ortholog *unc-51* in *C. elegans*, which display uncoordinated movement with an underlying axonal defect [22–28]. A neuronal function of ULK orthologs was subsequently also reported in *Drosophila*, zebrafish and mammals [27, 29–33]. This autophagy-independent function of ULK proteins does not appear to involve other canonical autophagy proteins, including components of the ATG1/ULK protein complex such as ATG13 and FIP200 [34, 35]. Instead, the neuronal function of ULK proteins is dependent on different sets of proteins that include – depending on the organism analyzed – UNC-14, VAB-8 and PP2A (*C. elegans*), UNC-76 (*Drosophila*), and Syntenin and SynGAP (mammals) several of which are phosphorylated by ULKs [26–28, 33, 36–41]. Thus, the two known functions of ULK proteins in autophagy and neuronal processes involve different sets of proteins.

Apoptosis-induced proliferation (AiP) is a specialized form of compensatory proliferation that occurs after massive cell loss due to stress-induced apoptosis [42–45]. Initially described in *Drosophila* where it can compensate for the apoptotic loss of up to 60 % of imaginal disc cells [46], AiP has since been observed in many organisms, including classical regeneration models such as hydra, planarians, zebrafish, xenopus, and mouse [47–50]. Interestingly, AiP is directly dependent on a non-apoptotic function of caspases that otherwise execute the apoptotic program in the dying cell. In *Drosophila*, the initiator caspase Dronc triggers activation of Jun-N-terminal kinase (JNK) signaling, which leads to the production of mitogens including Wingless (Wg), Decaplentaplegic (Dpp), and the EGF ligand Spitz for AiP [51–59].

**Fig. 1:**
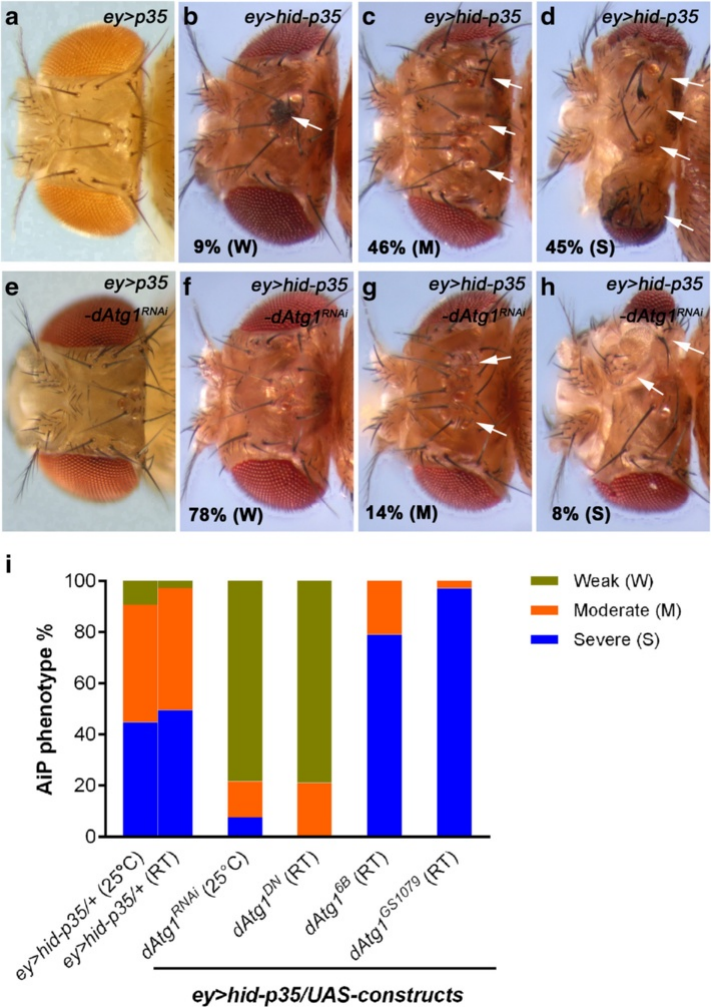
Suppression of *ey > hid-p35* by loss of *dAtg1*. The hyperplastic overgrowth phenotype of *ey > hid-p35* can be grouped in three categories, weak (W, including wildtype-like), moderate (M) and severe (S), as previously described [52]. Moderate flies are characterized by overgrowth of head capsules with duplications of bristles (arrows) and ocelli (arrowhead), while severe flies have overgrown and deformed heads with amorphic tissue. Each screen analysis was repeated at least twice with scoring more than 50 *ey > hid-p35/*(RNAi or mutant) adult flies. **a**–**h** Representative dorsal views of adult fly head capsules of the indicated genotypes. **a**–**d** Compared to the control *ey > p35*, which is similar to wildtype (**a**), percentages of *ey > hid-p35* flies display weak (**b**), moderate (**c**) and severe (**d**) phenotypes (9 %, 46 %, and 45 %, respectively). Therefore, over 90 % of *ey > hid-p35* flies show a clear hyperplastic overgrowth phenotype (either severe or moderate). **e** Knockdown of *dAtg1* by RNAi in *ey > p35* does not cause obvious defects on head capsules. **f**–**h** *dAtg1* RNAi strongly reduces the percentage of *ey > hid-p35* flies showing severe (8 %) and moderate (14 %) overgrowth phenotype and largely extends the population of flies with a weak or wildtype-like appearance (78 %). **i** Summary of the suppression of the *ey > hid-p35* overgrowth phenotype by expressing *dAtg1* ^*RNAi*^ or dominant-negative *dAtg1* ^*DN*^ and the enhancement of the phenotype by expressing two constructs encoding *dAtg1* (*dAtg1* ^*6B*^ and *dAtg1* ^*GS1079*^). Either 25 °C or room temperature (RT, 22 °C) was used for these analyses. The majority of *ey > hid-p35* flies (*ey > hid-p35/+*) display either severe or moderate overgrowth phenotypes at both 25 °C and RT. Blue indicates severe, orange indicates moderate, and green indicates weak or wildtype-like phenotypes

However, many mechanistic details of AiP are still unknown. Therefore, we and others have developed several AiP models in eye and wing imaginal discs in *Drosophila* [52, 57, 58, 60–64]. In the first set of AiP models, apoptosis is induced upstream by expression of cell death-inducing factors such as *hid* or *reaper*, but blocked downstream by co-expression of the effector caspase inhibitor *p35*, generating ‘undead’ cells [52, 55, 56, 62]. Because undead cells do not die and P35 does not inhibit the initiator caspase Dronc, Dronc continues to generate the signals for AiP, which causes tissue overgrowth. For example, *ey-Gal4*-driven *UAS-hid* and *UAS-p35* (*ey > hid-p35*) cause overgrowth of head capsules with ectopic sensory organs such as bristles and ocelli, and in severe cases forms amorphic head tissue (Fig. 1a–d) [52]. The *ey > hid-p35* undead model is the only known overgrowth-promoting AiP model in which adult animals survive [52]. Other undead AiP models, mostly in the wing, such as *nub > hid-p35* or *hh > hid-p35* produce enlarged larval wing imaginal discs, but do not allow adult animals to eclose. Thus, the *ey > hid-p35* undead AiP model is a convenient tool for genetic screening to identify genes involved in AiP by scoring adult flies.

The second type of AiP models does not involve the use of p35 and has been referred to as genuine or regenerative AiP [43, 52, 60, 61, 63]. These models take advantage of Gal80^ts^, a temperature-sensitive inhibitor of Gal4, which allows temporal control of UAS-transgene expression by a temperature shift to 29 °C [65]. Because these AiP models are p35-independent, cells complete the apoptotic program and we score the ability of the affected tissue to regenerate the lost cells by new proliferation. In our genuine/regenerative AiP model, we express the pro-apoptotic factor *hid* for 12 h under control of *dorsal-eye-Gal4* [66] (referred to as *DE*^*ts*^*-hid*) in eye imaginal discs during second or early third larval instar [52]. This treatment causes massive tissue loss which is regenerated by AiP within 72 h after tissue loss.

Here, we report the identification of *dAtg1* as a suppressor of the overgrowth phenotype of the undead *ey > hid-p35* AiP model. *dAtg1* is also required for complete regeneration in the *DE*^*ts*^*-hid* AiP model. Furthermore, we show that *dAtg1* is genetically acting downstream of JNK activation, but upstream of mitogen production such as Wg. Consistently, *dAtg1* is transcriptionally induced by JNK activity during AiP. Interestingly, the involvement of *dAtg1* in AiP is independent of other *dAtg* genes, including *dAtg13*, *dAtg17*/*Fip200*, *dAtg6*, *vps15*, *vps34*, *dAtg7*, and *dAtg8*. These findings suggest that *dAtg1* has an autophagy-independent function in AiP. Finally, *dAtg1* is not employing the mechanism used during neuronal development as targeting *unc-76* did not affect AiP. Therefore, in addition to a role of *dAtg1* in autophagy and neuronal development, we define a third function of *dAtg1* for AiP.

## Results

### *dAtg1* is a suppressor of apoptosis-induced proliferation

AiP phenotypes of *ey > hid-p35* animals vary from mild to moderate to severe overgrowth of head capsules characterized by pattern duplications of ocelli, bristles, and sometimes entire antennae (moderate) as well as deformed heads with amorphic tissue (severe) (Fig. 1a–d) [52]. To identify genes required for AiP, we are screening for suppressors of the *ey > hid-p35*-induced overgrowth phenotypes. For follow-up characterization of identified suppressors, we are using undead and regenerative (p35-independent) AiP models in eye and wing imaginal discs.

Using this approach, we identified *dAtg1* as a strong AiP suppressor of *ey > hid-p35* by RNAi (Fig. 1f–h). The percentage of *ey > hid-p35* animals with severe and moderate AiP phenotypes is strongly reduced upon *dAtg1* knock-down (Fig. 1f–h; quantified in Fig. 1i). No effect was scored on control (*ey* > *p35*) animals (Fig. 1e). Although *dAtg1* RNAi results in significant loss of dATG1 mRNA and protein levels (Additional file 1: Figure S1A–B’) and no off-targets have been reported, we tested additional reagents for an involvement of *dAtg1* in AiP. Expression of a dominant negative *dAtg1* transgene also suppressed *ey > hid-p35*-induced overgrowth (Fig. 1i). Furthermore, increased expression of *dAtg1*, which does not alter apoptosis (Additional file 2: Figure S2), enhances the AiP phenotype and generates many animals with severe AiP phenotype (Fig. 1i). We therefore conclude that *dAtg1* is required for tissue overgrowth in the undead AiP model.

### *dATG1* is required for regenerative apoptosis-induced proliferation

**Fig. 2:**
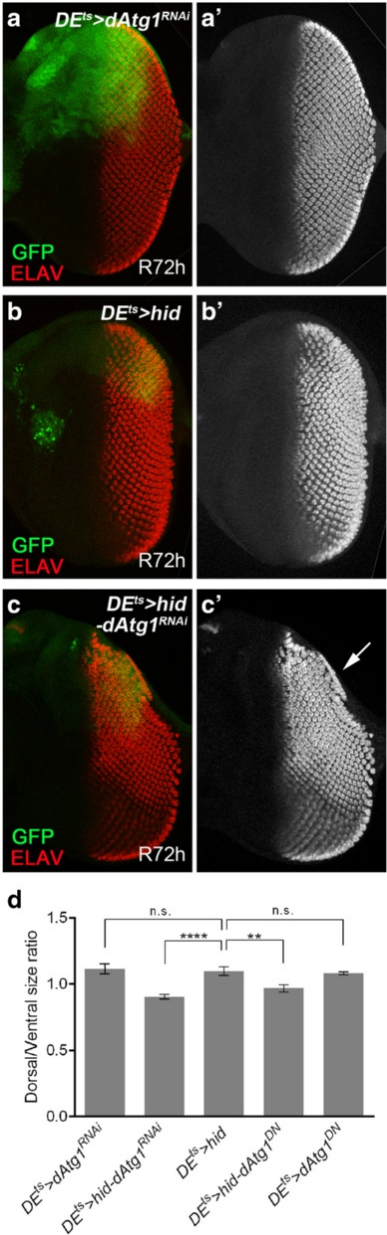
*dAtg1* is required for complete tissue regeneration in response to apoptosis. **a**–**c’** Late third instar eye discs, anterior is to the left. ELAV labels photoreceptor neurons and is used to outline the shape of the posterior part of the discs. Conditional expression of *dAtg1* ^*RNAi*^ (**a**, **a’**), *hid* (**b**, **b’**), or *hid* and *dAtg1* ^*RNAi*^ (**c**, **c’**) was under control of *DE-Gal4* and *tub-Gal80* ^*ts*^ (*DE* ^*ts*^) and indicated by GFP. A temperature shift to 29 °C for 12 h during second instar larval stage induced expression of these transgenes which is followed by a recovery period of 72 h at 18 °C (R72h). (**a**, **a’**) Following such a temperature shift procedure, expression of *dAtg1* ^*RNAi*^ alone (*DE* ^*ts*^ *> dAtg1* ^*RNAi*^) does not affect the eye disc morphology indicated by the normal ELAV pattern in the dorsal half of the eye disc (red in a, grey in a’). (**b**, **b’**) *DE* ^*ts*^ *> hid* induced massive apoptosis (GFP puncta and aggregates, arrow in **b**), which results in loss of bilateral symmetry of the disc 24 h after the temperature shift [52]. However, as indicated by the largely normal ELAV pattern in late third instar eye discs, the apoptosis-induced tissue damage has fully recovered after 72 h recovery (R72h) at 18 °C. (**c**, **c’**) A *DE* ^*ts*^ *> hid* eye disc that was simultaneously treated with *dAtg1* ^*RNAi*^ (*DE* ^*ts*^ *> hid-dAtg1* ^*RNAi*^). The arrow in (**c’**) highlights the incomplete ELAV pattern on the dorsal half of the disc indicating that the regenerative response was partially impaired by reduction of *dAtg1*; 79 % (n = 28) of *DE* ^*ts*^ *> hid-dAtg1* ^*RNAi*^ eye discs showed incomplete regeneration. (**d**) Quantification of the dorsal/ventral size ratio (mean ± SE) in eye discs of various genotypes. One-way ANOVA with Bonferroni multiple comparison test was used to compute *P* values. Asterisks indicate a statistically significant change on dorsal/ventral size ratio compared to the control *DE* ^*ts*^ *> hid*. Compared to *DE* ^*ts*^ *> hid*, expression of *dAtg1* ^*RNAi*^ or *dAtg1* ^*DN*^ significantly (****P < 0.0001 and **P < 0.01, respectively) reduces the size of the dorsal half of the eye disc. As the controls, disc sizes of *DE* ^*ts*^ *> dAtg1* ^*RNAi*^ and *DE* ^*ts*^ *> dAtg1* ^*DN*^ are not significantly (n.s.) different from those of *DE* ^*ts*^ *> hid*

Encouraged by the identification of *dAtg1* in the undead AiP model, we examined an involvement of *dAtg1* in the regenerative (*p35*-independent) *DE*^*ts*^ > *hid* AiP model. When *hid* expression is induced for 12 h in the dorsal half of the eye imaginal disc, the dorsal half of the eye disc is severely ablated [52]. After 72 h recovery (R72h), the disc has recovered due to regenerative growth by AiP (Fig. 2b) [52]. The degree of the regenerative response can be easily assessed by visualization of the photoreceptor pattern using ELAV as a marker, because photoreceptor differentiation follows tissue growth of the disc [67]. *DE*^*ts*^ > *hid* control discs regenerate a normal ELAV pattern 72 h after *hid*-induced tissue ablation (Fig. 2b, b’). In contrast, *DE*^*ts*^ > *hid* imaginal discs expressing *dAtg1*^*RNAi*^ are unable to fully regenerate the ablated tissue (Fig. 2c). The ELAV pattern in the dorsal half of the eye disc is incomplete (arrow in Fig. 2c’), suggesting that the regenerative response after *hid*-induced tissue ablation is partially blocked by *dAtg1* RNAi. As additional control, *DE*^*ts*^ > *dAtg1*^*RNAi*^ alone does not affect the ELAV pattern (Fig. 2a, a’). These findings are also confirmed by expression of a dominant negative *dAtg1* transgene and quantified in Fig. 2d. In summary, these data illustrate that *dAtg1* is an important gene required for AiP in both undead and regenerative models. We also considered examining the effect of overexpressed *dAtg1* in regenerative AiP. However, expression of *dAtg1* alone using the *DE-Gal4* driver triggers strong apoptosis (Additional file 2: Figure S2C), consistent with a previous report [6], which may complicate the interpretation of the results. Therefore, we did not characterize the role of overexpressed *dAtg1* in the regenerative AiP model.

### *dAtg1* is required for AiP downstream of Dronc in undead eye and wing imaginal discs

**Fig. 3:**
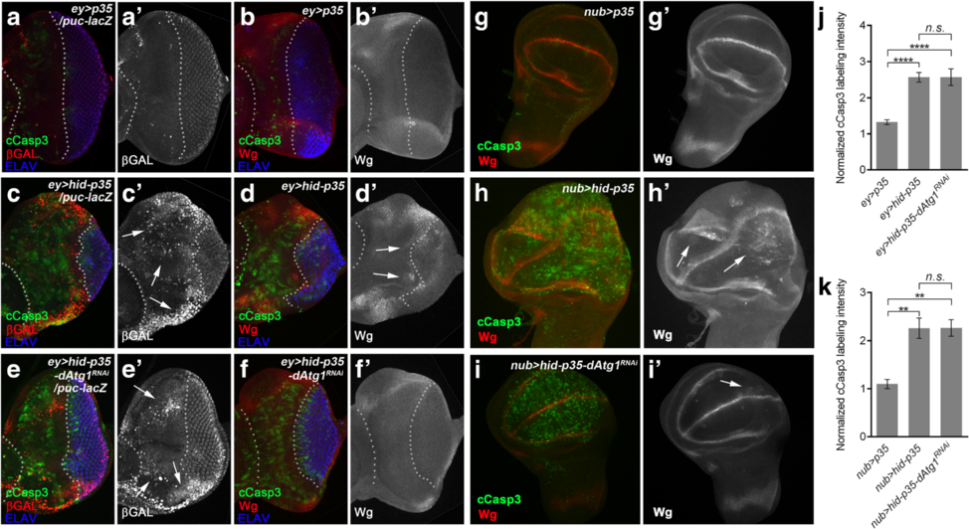
*dAtg1* acts genetically downstream of or in parallel to JNK and upstream of Wg expression. Late third instar eye (**a**–**f’**) or wing (**g**–**i’**) discs, anterior is to the left. The cleaved Caspase-3 (cCasp3) labeling (green in **a**–**i**) indicates activity of Dronc in *p35*-expressing tissues. White dotted lines in (**a**–**f’**) indicate the anterior portion of the eye discs which expresses *ey-Gal4*. ELAV labels photoreceptor neurons (blue in **a**–**f**) and is used to mark the posterior differentiating eye field. (**a**–**b’**) In *ey > p35* control discs, *puc-lacZ* expression (β-Gal; red in **a**, grey in **a’**) as a marker of JNK/Bsk activity is low (**a’**, arrow) in the anterior portion of the eye discs. Expression of Wg (red in **b**, grey in **b’**) is restricted to dorsal and ventral edges of the eye discs. Dronc activity indicated by cCasp3 labeling is low. (**c**–**d’**) In *ey > hid-p35* discs, Dronc activity (cCasp3 labeling) is strongly induced in undead anterior tissue (**c**, **d**). The anterior portion of the discs between the white dotted lines is significantly expanded and displaces the eye field in the posterior portion of the discs (ELAV). Compared to the *ey > p35* control discs (**a’**, **b’**), in the overgrown anterior eye portion, JNK activity (**c’**, arrows) and expression of Wg (**d’**, arrows) are strongly induced. (**e**–**f’**) Expression of *dAtg1* ^*RNAi*^ suppresses hyperplastic overgrowth in about 80 % of the *ey > hid-p35-dAtg1* ^*RNAi*^ discs (n > 60) indicated by the normalized ELAV pattern. This ratio corresponds to the suppression of the adult overgrowth phenotype (Fig. 1i). However, *puc-lacZ* expression and cCasp3 labeling are not suppressed by *dAtg1* ^*RNAi*^ (**e’**, arrows) in contrast to ectopic Wg expression, which is blocked (**f’**) in the anterior portion of the eye discs. (**g**–**i’**) Compared to the control wing discs where *p35* is expressed in the pouch area under the control of *nub-Gal4* (*nub > p35*; **g**, **g’**), in *nub > hid-p35* discs, co-expression of *hid* and *p35* induces tissue overgrowth, increased cCasp3 labeling, and ectopic Wg expression (**h**, **h’**; arrows). Similar to eye discs, expression of *dAtg1* ^*RNAi*^ largely blocks tissue overgrowth and ectopic Wg, but not the cCasp3 labeling (**i**, **i’**). A low level of ectopic Wg remains in *nub > hid-p35-dAtg1* ^*RNAi*^ discs (**i’**, arrow). (**j, k**) Quantification of cCasp3 labeling intensity in eye and wing discs (mean ± SE). *dAtg1* RNAi has no obvious effects on the cCasp3 labeling induced by expression of *hid* and *p35* in both eye (**j**) and wing (**k**) discs

We further characterized the role of *dAtg1* for AiP with molecular markers. Although *dAtg1* is best characterized for a role in autophagy, it is theoretically possible that *dAtg1* RNAi inhibits apoptosis and thus AiP indirectly. Therefore, we first tested how *dAtg1* relates genetically to Dronc in the AiP pathway. As a marker for Dronc activity, we used the cleaved Caspase-3 (cCasp3) antibody. Although apoptosis is blocked by *p35* expression, the cCasp3 antibody still labels undead cells (Fig. 3c, d), presumably because Dronc also has non-apoptotic substrates [52, 68]. *dAtg1* RNAi suppresses the overgrowth and normalizes the morphology of the *ey > hid-p35* eye disc as judged by ELAV labeling (Fig. 3e, f). However, cCasp3 labeling is not significantly altered by *dAtg1* RNAi (Fig. 3e, f, j) despite the rescue of disc morphology suggesting that the loss of *dAtg1* does not affect caspase activity in undead tissues.

We also characterized the involvement of *dAtg1* in AiP in undead wing imaginal discs. Expression of *hid* and *p35* under *nub*-*Gal4* control (*nub > hid-p35*) causes strong overgrowth of the wing imaginal disc compared to *nub* > *p35* control discs (Fig. 3g, h). *dAtg1* RNAi suppresses the overgrowth of *nub > hid-p35* wing discs, but leaves cCasp3 activity intact (Fig. 3i, k). To further confirm these data obtained by RNAi, we conducted mosaic analysis using *dAtg1* null mutants in wing imaginal discs because homozygous *dAtg1* mutants are early larval lethal in the *ey > hid-p35* genetic background. Consistent with RNAi results, *dAtg1* mutants do not alter cCasp3 labeling induced by co-expression of *hid* and *p35* in MARCM clones (Additional file 3: Figure S3A–C). Similarly, *dAtg1* mutant clones or RNAi do not suppress *GMR-hid*-induced apoptosis in the eye (Additional file 3: Figure S3D–F). Together, these data further confirm that loss of *dAtg1* does not affect apoptosis and that *dAtg1* controls AiP downstream of caspase (Dronc) activation.

### *dAtg1* is required for AiP downstream of JNK, but upstream of *wingless* in undead eye and wing imaginal discs

Next, because JNK is an important mediator of AiP [43, 52, 56, 57], we determined the position of *dAtg1* relative to JNK in the AiP pathway. The JNK activity reporter *puc-lacZ* is strongly induced in AiP models compared to controls (Fig. 3a’, c’; arrows) [52, 54, 56, 57]. The morphology of the discs is severely disrupted, which correlates with signal intensity of *puc-lacZ*, especially in overgrown areas. In response to *dAtg1* RNAi, overgrowth and disc morphology, as judged by ELAV labeling, is restored to almost normal (Fig. 3e). Nevertheless, despite the rescue of disc morphology, *puc-lacZ* expression is not significantly reduced (Fig. 3e’; arrows). These data suggest that *dAtg1* acts downstream of or in parallel to JNK activity in the AiP pathway.

Finally, we determined the position of *dAtg1* relative to *wingless* (*wg*), another marker in the AiP pathway [54–56]. Wg and its orthologs are critical mediators of AiP in regenerative responses in many animals (reviewed by [43, 45]). In undead eye discs, inappropriate *wg* expression is induced compared to controls (Fig. 3b, b’, d, d’; arrows). *dAtg1* knockdown normalizes *wg* expression in the disc (Fig. 3f, f’). In addition, in undead *nub > hid-p35* wing imaginal discs, *wg* expression is strongly induced (arrows in Fig. 3h’). However, similar to undead eye discs, co-expression of *dAtg1* RNAi in *nub > hid-p35* discs suppresses overgrowth (Fig. 3i) and normalizes the *wg* pattern (Fig. 3i’). Together, these analyses suggest that *dAtg1* acts genetically downstream of Dronc and either downstream of or in parallel to JNK, but upstream of Wg, in the AiP network.

**Fig. 4:**
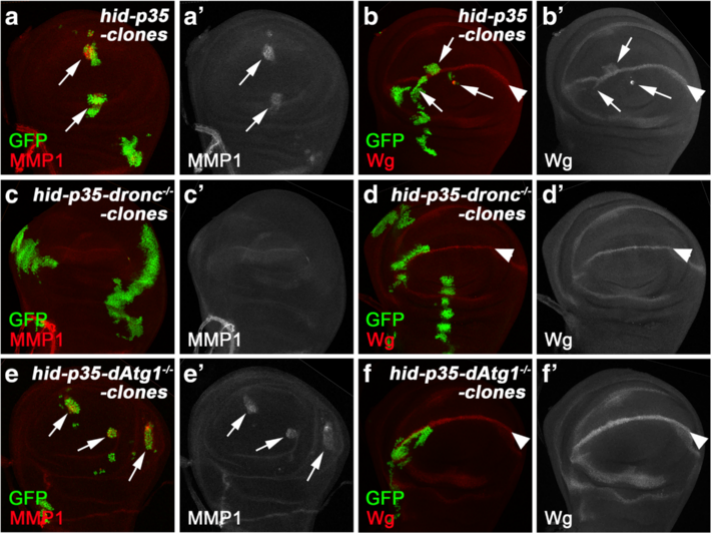
*dAtg1* is required cell autonomously for Wg expression, but not JNK activation, in undead clones. Late third instar wing discs with mosaic clones positively marked by GFP, anterior is to the left. MMP1 labeling (red in **a**, **c**, **e** and grey in **a’**, **c’**, **e’**) is used as marker of JNK activity. Wg (red in **b**, **d**, **f** and grey in **b’**, **d’**, **f’**) is highly expressed at the dorsal/ventral (D/V) boundaries (arrowheads in **b**, **d**, **f**) of wing discs. (**a**–**b’**) Simultaneous expression of *hid* and *p35* in clones. MMP1 expression (arrows in **a**, **a’**) is induced in all *hid* and *p35* co-expressing clones. Ectopic expression of Wg (arrows in **b**, **b’**) was observed in over 80 % of clones (n = 66) generated in close proximity to the D/V boundaries in the wing discs. Genotype: *hs-FLP tub-GAL4 UAS-GFP/UAS-hid; UAS-p35/+; tub-GAL80 FRT80B/FRT80B*. (**c**–**d’**) Simultaneous expression of *hid* and *p35* in *dronc* mutant clones. Both MMP1 labeling and ectopic Wg expression, induced by co-expression of *hid* and *p35*, are completed blocked in *dronc* mutant clones (n > 30). Genotype: *hs-FLP tub-GAL4 UAS-GFP/UAS-hid; UAS-p35/+; tub-GAL80 FRT80B/droncI29 FRT80B*. (**e**–**f’**) Simultaneous expression of *hid* and *p35* in *dAtg1* mutant clones. *hid* and *p35*-induced MMP1 expression persists in *dAtg1* mutant clones (arrows in **e**, **e’**). In contrast, the ectopic Wg expression induced by *hid* and *p35* is suppressed in over 70 % of *dAtg1* clones (n = 73) generated in close proximity to the D/V boundaries in the wing discs. Genotype: *hs-FLP tub-GAL4 UAS-GFP/UAS-hid; UAS-p35/+; tub-GAL80 FRT80B/dAtg1* ^*Δ3D*^ *FRT80B*

In addition to the RNAi analysis, we also co-expressed *hid* and *p35* in either wildtype, *dronc*, or *dAtg1* mutant clones (by MARCM) and examined for JNK activity (using MMP1 as JNK marker [69]) and Wg expression (Fig. 4a, a’, b, b’). Ectopic Wg expression is most frequently observed in the wing pouch area in close proximity to the dorsoventral boundary in the wing disc (Fig. 4b; arrows), similar to previous reports [56]. The induction of MMP1 and Wg expression is dependent on Dronc as co-expression of *hid* and *p35* in *dronc* mutant clones suppresses these AiP markers (Fig. 4c–d’). Importantly, when *hid* and *p35* were co-expressed in *dAtg1* mutant clones, the expression of Wg was suppressed, while MMP1 expression was not affected (Fig. 4e–f’) suggesting that *dAtg1* acts downstream of or in parallel to JNK activity, but upstream of Wg. These data are consistent with the RNAi data (Fig. 3).

Because *dAtg1* is required for *wg* expression in AiP, we tested if *dAtg1* was also sufficient for expression of AiP markers including *wg*, *dpp*, and *kekkon1* (*kek*), the latter being a marker of EGFR activity [51, 52, 54–56, 70]. However, while expression of *hid* in the *DE*^*ts*^ > *hid* model is sufficient to induce *wg*, *dpp*, and *kek* expression (Additional file 4: Figure S4A–B’, D–E’, G–H’), expression of *dAtg1* alone under the same conditions (*DE*^*ts*^ > *Atg1*) is not (Additional file 4: Figure S4C, C’, F, F’, I, I’). These observations suggest that, in addition to *dAtg1* expression, additional caspase-dependent events have to occur in order to induce AiP.

### *dAtg1* is transcriptionally induced for AiP in a JNK-dependent manner

**Fig. 5:**
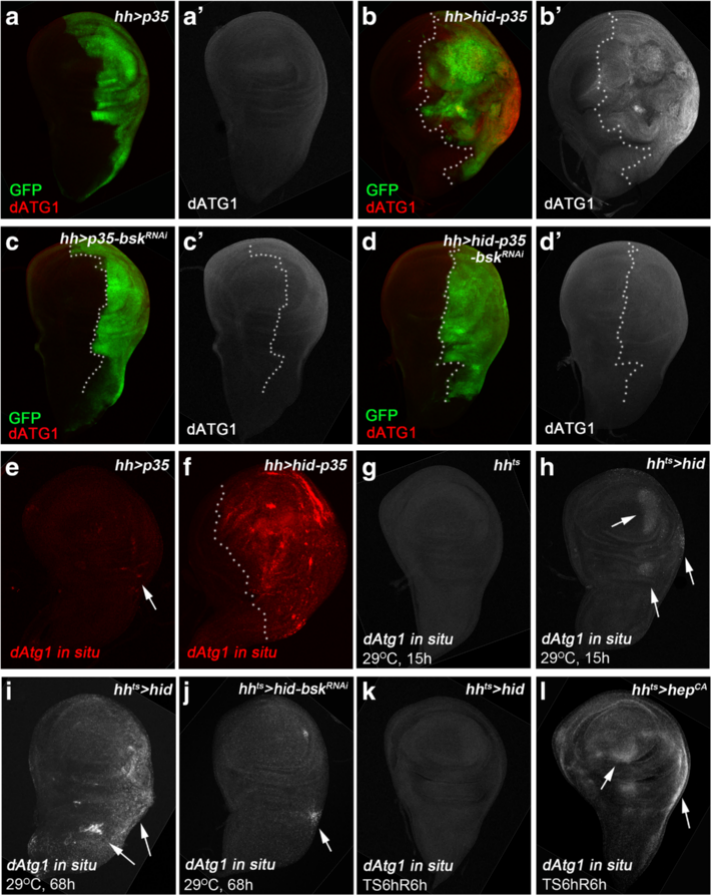
*dAtg1* is transcriptionally induced for AiP in a JNK-dependent manner. Late third instar wing discs, anterior is to the left. White dotted lines indicate the anterior/posterior compartment boundaries. *hh-Gal4* is used to drive expression of various transgenes in the posterior compartment of wing discs. (**a**–**d’**) Wing discs are labeled with dATG1 (red in **a**, **b**, **c**, **d** and grey in **a’**, **b’**, **c’**, **d’**). GFP marks the posterior disc compartment where *hh-Gal4* is expressed (green in **a**, **b**, **c**, **d**). Compared to *hh > p35* controls (**a**, **a’**), co-expression of *hid* and *p35* by *hh > Gal4* induces overgrowth of the posterior wing compartment as indicated by enlarged tissue size and folded disc morphology (**b**, **b’**). dATG1 protein is strongly increased in the overgrown posterior tissue (compare **b’** to **a’**). Knockdown of JNK (*bsk* ^*RNAi*^) has no effect on dATG1 expression in the control *hh > p35* discs (**c**, **c’**), but it suppresses overgrowth as well as accumulation of dATG1 in *hh > hid-p35* discs (compare **d**, **d’** to **b**, **b’**). (**e**–**l**) Wing discs labeled with *dAtg1* in situ antisense probes (red in **e**, **f** and grey in **g**–**l**). (**e**, **f**) Compared to the control (**e**), *dAtg1* transcription, as indicated by the fluorescent in situ signals of *dAtg1*, is increased in *hh > hid-p35* discs (**f**). (**g**–**l**) *hh-Gal4 tub-Gal80* ^*ts*^ (*hh* ^*ts*^) was used to control temporal expression of GAL4 alone as the control (**g**), *hid* (**h**, **i**, **k**), *hid* and *bsk* ^*RNAi*^ (**j**), or a constitutively activated form of JNK kinase, *hep* ^*CA*^ (**l**). A weak increase of *dAtg1* transcript was observed in the posterior wing tissues after a 15 h expression of *hid* (**h**, arrows). *dAtg1* transcript is strongly increased after *hid* expression for 68 h (**i**, arrows). This increase of *dAtg1* transcripts is inhibited by knockdown of JNK (*bsk* ^*RNAi*^) with only a low level of *dAtg1* induction left in *hh* ^*ts*^ *> hid,bsk* ^*RNAi*^ discs (**j**, arrow, compared to **i**). Although expression of *hid* at 29 °C for 6 h followed by recovery at 18 °C for 6 h (TS6hR6h) does not trigger accumulation of *dAtg1* (**k**), expression of *hep* ^*CA*^ (to activate JNK) under the same condition is sufficient to induce expression of *dAtg1* (**l**, arrows)

Next, we examined if protein and transcript levels of *dAtg1* change in AiP. Indeed, using a dATG1-specific antibody (Additional file 1: Figure S1B, C) [71], we observed increased protein abundance of dATG1 in the undead compartment of wing discs compared to controls (Fig. 5a, b). To determine if this is a transcriptional or translational effect on dATG1 levels in undead cells, we performed mRNA in situ hybridization assays on undead (*hh > hid-p35*) and regenerative (*hh*^*ts*^ > *hid*) wing imaginal discs. In both AiP models, *dAtg1* is transcriptionally induced (Fig. 5e–i). Additional file 5: Figure S5 demonstrates the specificity of the *dAtg1* in situ probes. The *hh*^*ts*^ > *hid* regenerative model allows determination of the timing of *dAtg1* expression during AiP. *dAtg1* expression is slow as a pulse of *hid* expression for 15 h only weakly induces it (Fig. 5h). Only after prolonged expression of *hid* (68 h), is a strong induction of *dAtg1* expression detectable (Fig. 5i). These data suggest that *dAtg1* expression occurs quite late in the AiP response.

Because *dAtg1* acts genetically downstream of or in parallel to JNK (Figs. 3 and 4) and because JNK can induce *dAtg1* expression under oxidative stress conditions and by ectopic activation of JNK [72], we tested if the transcriptional induction of *dAtg1* in the AiP models is also dependent on JNK. The *Drosophila* JNK homolog is encoded by the gene *basket* (*bsk*) [73, 74]. Indeed, while *bsk* RNAi does not affect *dAtg1* expression in control discs (Fig. 5c, c’), it suppresses the accumulation of dATG1 protein in undead and *dAtg1* transcripts in regenerative wing discs (Fig. 5d, d’, j). Consistent with a previous report [72], ectopic JNK activation by expression of a constitutively active JNKK transgene (*hep*^*CA*^) for a short pulse of 6 h with 6 h recovery at 18 °C (TS6hR6h) is sufficient to induce *dAtg1* expression in wing imaginal discs (Fig. 5l). However, expression of the pro-apoptotic gene *hid* under the same conditions (TS6hR6h) cannot induce *dAtg1* expression (Fig. 5k). Combined, these data suggest that *dAtg1* expression is under direct control of JNK signaling, while it is far downstream of Hid expression.

### Undead tissue produces autophagosome-like particles which do not contribute to apoptosis-induced proliferation

*dAtg1* acts upstream in the autophagy pathway and its activation can induce autophagy [6, 10, 17]. Oxidative stress or ectopic activation of JNK has been previously reported to induce expression of multiple *dAtg* genes, including *dAtg1*, as well as autophagy in midgut and fat body cells [72]. We therefore examined if autophagy is induced in undead disc tissue and whether it contributes to AiP. Because dATG8 is an essential part of autophagosomes, fusion proteins of dATG8 with fluorescent proteins such as GFP or mCherry are used as markers for formation of autophagosomes [7]. Moreover, because GFP is stable in autophagosomes, but unstable in autolysosomes, whereas mCherry is stable in both compartments, the tandem fusion protein GFP-mCherry-dATG8a is used as marker for the maturation of autophagosomes into autolysosomes, indicating autophagic flux [75, 76]. Indeed, as shown in Additional file 6: Figure S6, undead *ey* > *hid*-*p35*-expressing tissue accumulates large quantities of GFP-mCherry-dATG8a-containing particles. However, it is unclear if these particles are classical autophagosomes. While the GFP signals are weaker compared to the mCherry signals, which may be an indicator of autophagic flux, there are clearly GFP-only particles which do not display mCherry fluorescence (compare Additional file 6: Figure S6b’ and S6b”). This observation is inconsistent with the concept of autophagic flux [75]. Furthermore, even though *dAtg1* RNAi suppresses AiP, it does not suppress the formation of the GFP-mCherry-dATG8a particles (Additional file 6: Figure S6C–C”). This result suggests that the ectopic expression of *dAtg1* in undead tissue does not induce the formation of the GFP-mCherry-dATG8a-containing particles. Furthermore, and more importantly, these particles do not contribute to the overgrowth of undead tissue nor, thus, to AiP.

### Other *dAtg* genes mediating autophagy and *unc-76* are not required for apoptosis-induced proliferation

**Fig. 6:**
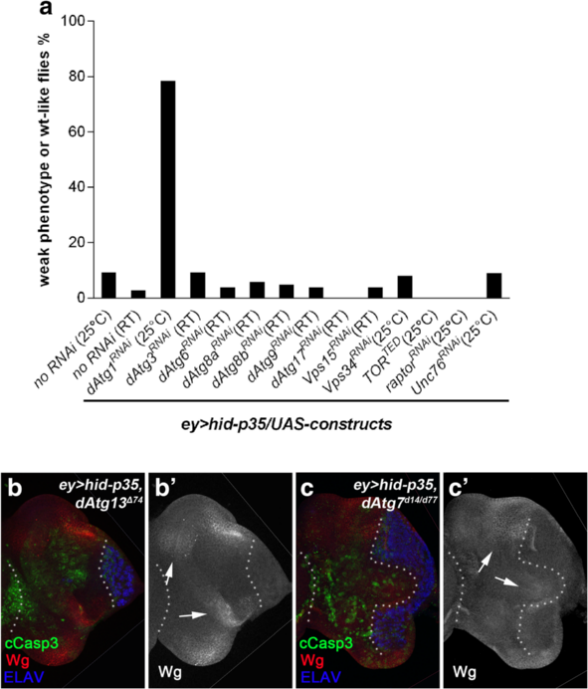
Key components of the autophagy pathway, other than *dAtg1*, do not modify the *ey > hid-p35* phenotype. (**a**) Results of the suppression of *ey > hid-p35* using RNAi targeting components of the autophagy pathway in *Drosophila*. Representative RNAi results for each gene were shown. Compared to the control where no RNAi was used, knockdown of *dAtg1* significantly increases the percentage of weak phenotype or wildtype-like *ey > hid-p35* flies to about 80 %. However, knockdown of *dAtg3*, *dAtg6*, *dAtg8a*, *dAtg8b*, *dAtg9*, *dAtg17*, *vps15*, and *vps34* does not suppress the *ey > hid-p35* overgrowth phenotype. In contrast, expression of a kinase dead form of TOR (TOR^TED^) or knockdown of *raptor*, both of which cause activation of *dAtg1*, enhances the AiP phenotype. However, RNAi targeting *unc-76*, which mediates the function of *dAtg1* in neuronal development, does not suppress the overgrowth of *ey > hid-p35* flies. Room temperature (RT) was used in some cases due to strong lethality caused by expressing these RNAi lines at 25 °C in the background of *ey > hid-p35*. The *ey > hid-p35* flies display comparable overgrowth phenotypes at 25 °C and RT. (**b**–**c’**) Late third instar eye discs labeled with cCasp3, Wg, and ELAV. Neither *dAtg13* null mutants (*dAtg13* ^*Δ74*^) (**b**, **b’**) nor *dAtg7* null mutants (*dAtg7* ^*Δ14/*^ *dAtg7* ^*Δ77*^) (**c**, **c’**) inhibit overgrowth, cCasp3 labeling and ectopic Wg expression (**b’**, **b’**; arrows) in *ey > hid-p35* discs (at least 40 discs were analyzed for each genotype)

Because of this unexpected result, we tested other *dAtg* genes for an involvement in AiP. Surprisingly, RNAi targeting *dAtg3*, *dAtg6*, *dAtg8a*, *dAtg8b*, *dAtg9*, and *dAtg17* as well as *vps15* and *vps34* had no effect on AiP (Fig. 6a). Most notable are *dAtg3* and *dAtg8* because they encode essential components for autophagosome maturation (see Background) [3, 19–21]. To ensure that the RNAi transgenes used to target these *dAtg* genes are functionally intact, we tested them in two functional assays. They suppressed starvation-induced autophagy in the fat body (Additional file 7: Figure S7A–E’) demonstrating that *dAtg3*, *dAtg8a*, and *dAtg8b* are efficiently knocked down to induce an autophagy-deficient phenotype. In addition, the functionality of these RNAi stocks is further confirmed in that they all enhanced the *eyeful* phenotype (Additional file 4: Figure S4F–J) which is known to be enhanced by loss of autophagy [77]. The *eyeful* (*ey*-*Gal4 UAS-Dl,psq,lola*) [78] condition uses the same *Gal4* driver as in the *ey > hid-p35* AiP model. Therefore, tissue-specific and/or Gal4-dependent differences do not account for the failure of these RNAi stocks to suppress AiP.

In addition to targeting essential autophagy components by RNAi, we also tested homozygous *dAtg13* and *dAtg7* mutants which can survive to pupal or adult stages, respectively, for suppression of AiP. *dAtg13* encodes a component of the ATG1/ULK protein complex, while *dAtg7* encodes the E1-conjugating enzyme for autophagosome maturation. However, *dAtg13* and *dAtg7* mutants fail to suppress the abnormal morphology of *ey > hid-p35* discs as visualized by ELAV labeling and the ectopic Wg expression (Fig. 6b, c). These results suggest that the tested *dAtg* genes, except *dAtg1*, are not required for AiP. An involvement of *dAtg1* in AiP is further confirmed by expression of a kinase dead form of TOR (*TOR*^*TED*^) [79], which activates *dAtg1* [7], or RNAi knockdown of Raptor, an adaptor protein required for TOR activation [80], both of which enhance AiP (Fig. 6a).

Finally, we also examined the possibility that *dAtg1* uses the same mechanism in AiP that it uses during neuronal development. However, RNAi targeting *unc-76*, which is an important mediator of the function of *dAtg1* during neuronal development [27], does not suppress the overgrowth phenotype of the undead *ey > hid-p35* AiP model (Fig. 6a). Three independent RNAi lines gave consistent results. Therefore, in addition to autophagy and neuronal development, our data define a third function of *dAtg1* for AiP.

## Discussion

In this paper, we show that the sole ULK ortholog in *Drosophila*, *dAtg1*, is required for AiP both in undead and regenerative models. We demonstrated that *dAtg1* acts downstream of JNK activity in AiP and is transcriptionally induced by JNK, consistent with a previous study on oxidation response [72]. Furthermore, *dAtg1* is required for the expression of Wg, a mitogen associated with AiP [51, 52, 54–56, 81]. Finally, our data provide evidence that the role of *dAtg1* in AiP is independent on its role in canonical autophagy.

It is generally assumed that the secreted mitogens Wg, Dpp, and Spitz promote the proliferation of surviving cells during AiP [43, 45]. The expression of these genes is under control of JNK activity. Until recently, it was unknown how JNK signaling promotes expression of these genes. However, very recently, it was reported that an enhancer element in the *wg* gene that drives expression of *wg* under regenerative conditions contains three AP-1 binding sites required for regeneration [81]. AP-1 is composed of the transcription factors Jun and Fos (Kayak in *Drosophila*), which are controlled by JNK activity. This observation suggests a direct way of *wg* expression by JNK-dependent AP-1.

How does dATG1 fit into the AiP network? Our genetic data suggest that *dAtg1* acts downstream of or in parallel to JNK. Furthermore, we placed *dAtg1* genetically upstream of *wg* expression. Therefore, *dAtg1* may act in at least two different ways in the AiP network. It may directly modulate the activity of the AP-1 transcriptional complex. An indirect mode of action is also possible in which dATG1 provides a permissive environment for AP-1 activity. However, *dAtg1* does not control all AP-1 activities. Expression of *puc-lacZ* and *MMP*-1 are not affected by *dAtg1*^*RNAi*^ and *dAtg1* mutants, respectively (Figs. 3 and 4). In contrast, *wg* expression is suppressed under these conditions. Therefore, of the known transcriptional targets of JNK and AP-1 during AiP (*puc*-*lacZ*, *MMP*-*1*, *dAtg1*, and *wg*), *dAtg1* affects only *wg* expression. Future work will address the mechanistic role of dATG1 for the control of AiP.

Although *dAtg1* is required for AiP, it is not sufficient. Overexpression of *dAtg1* using *DE*^*ts*^-*Gal4* for 12 h followed by 24 h recovery does not trigger AiP markers such as *wg*-*lacZ*, *dpp*-*lacZ*, or *kek*-*lacZ* (Additional file 4: Figure S4). Expression of *hid* under the same conditions is able to induce these markers ectopically. These observations suggest that, in addition to *dAtg1* expression, apoptotic signaling triggers an additional activity required for *wg* expression and AiP.

The best characterized function of dATG1 and of ULKs in general is the initiation of autophagy under starvation or stress conditions [1, 2, 5, 10, 72]. Autophagy requires a total of 36 *Atg* genes [3]. Although we did not test all 36 *dAtg* genes for a role in AiP, we tested several genes which are critical for autophagy, including *dAtg3*, *dAtg6*, *dAtg7*, *dAtg8*, *dAtg9*, *dAtg13*, *dAtg17*, and *vps34. dAtg13* and *dAtg17* (aka *Fip200*) encode subunits of the ATG1/ULK complex [10–12]. ATG6 and VPS34 are subunits of the ATG6/Beclin complex, which is activated by ATG1 during autophagy. Phosphorylation of ATG9, the mammalian ortholog of dATG9, by ULK1 is required for autophagy [16, 17]. Finally, lipidation of ATG8, which is essential for formation of autophagosomes requires the function of ATG3 and ATG7 [3, 20, 21]. In contrast to *dAtg1*, inactivation of any of these genes does not suppress the overgrowth phenotype of *ey > hid-p35* animals. Furthermore, although we detect the formation of ATG8a-containing particles in undead eye imaginal discs, these particles are not dependent on *dAtg1* and do not contribute to AiP and overgrowth (Additional file 6: Figure S6). Combined, these data suggest that dATG1 does not trigger canonical autophagy in an AiP context.

In addition to autophagy, ULK proteins have also been implicated in neuronal development, most notably axon guidance and axonal growth [27, 30]. However, we also exclude a neuronal function of *dAtg1* in AiP because inactivation of *unc-76*, a mediator of dAtg1 for neuronal development [27], does not suppress overgrowth induced by *ey > hid-p35*.

## Conclusions

We revealed a third function of *dAtg1* in *Drosophila* for the control of regenerative proliferation after massive apoptotic cell loss. Future work will address if this role of *dAtg1* in regenerative proliferation is also conserved in other organisms, the molecular mechanism of this function, and whether it is potentially misregulated in pathological conditions such as cancer.

## Methods

### Fly strains and the *ey > hid-p35* assay

*UAS-dAtg1*^*[KQ#5B]*^ or *UAS-dAtg1*^*[K38Q]*^ were used to express an *dAtg1* kinase-dead mutant that functions as a dominant negative [6]. Either *UAS-dAtg1*^*6B*^ or *UAS-dAtg1*^*GS10797*^ were used to express wildtype *dAtg1* [6]. Both constructs gave similar results under the control of *Gal4* lines tested in this study. *Dorsal Eye-Gal4* (*DE-Gal4*) [66], *dronc*^*I29*^ [82], *dAtg1*^*Δ3D*^ [7], *dAtg13*^*Δ7*^ [10], *dAtg7*^*d14*^, *dAtg7*^*d77*^ [83], *dAtg8a*::*GFP-mCherry-dAtg8a* [76], and *eyeful* (*ey-Gal4 > UAS-delta*, *GS88A8 UAS-lola and UAS-pipsqueak*) [78] were as described. *puc-lacZ*^*E69*^, *wg-lacZ*, *dpp-lacZ*, *kek-lacZ*, *ey-Gal4*, *hh-Gal4*, *nub-Gal4*, *GMR-Gal4*, *tub-Gal80*^*ts*^, *UAS-p35*, *UAS-hid*, *UAS-hep*^*CA*^, *UAS-GFP*, and *UAS-TOR*^*TED*^ were obtained from the Bloomington Stock Center. UAS-based RNAi stocks of the following genes were obtained from Bloomington, VDRC or NIG-FLY stock centers: *bsk* (*BL 32977*, *V34138*)*, dAtg1* (*BL26731*), *dAtg3* (*BL34359*, *V101364*), *dAtg6* (*V22122*, *V110197*), *dAtg8a* (*V43076*, *V43097*), *dAtg8b* (*V17097*), *dAtg9* (*V10045*), *dAtg17* (*V106176*), *Vps15* (*V110706*, *NIG9746R-2*), *Vps34* (*V100296*), *raptor* (*BL34814*, *BL41912*), and *unc-76* (*V20721*, *V20722*, *V40495*). Comparable results were obtained from multiple RNAi lines targeting the same gene. Functionality of BL26731, V101364, V43097 and V17097 was tested on inhibition of starvation-induced autophagy [7] (Additional file 7: Figure S7). The exact genotype of *ey > hid-p35* is either *UAS*-*hid*; *ey*-*Gal4 UAS*-*p35* (*UAS-hid* on X; *ey-Gal4 UAS-p35* on second chromosome) or *UAS*-*hid*; *ey*-*Gal4 UAS*-*p35* (*UAS-hid* on X; *ey-Gal4 UAS-p35* on third chromosome; only used in Fig. 6c, c’). For analysis of *ey > hid-p35* adult hyperplastic phenotype, three categories, weak (W), moderate (M) and severe (S), were used as previously described [52]. Each screen analysis was repeated at least twice at 25 °C, or at room temperature (RT, 22 °C) if strong lethality was caused by expressing RNAi or dominant-negative mutant constructs at 25 °C in the background of *ey > hid-p35*, with scoring more than 50 *ey > hid-p35*/(RNAi or mutant) adult flies.

### Temperature-sensitive regenerative assays and statistical analysis

Larvae of the following genotypes (1) *DE*^*ts*^ *> hid* (*UAS-hid/+; UAS-GFP/+; DE-Gal4 tub-Gal80*^*ts*^*/+*); (2) *DE*^*ts*^ *> dAtg1*^*RNAi*^ (*UAS-GFP/+; DE-Gal4 tub-Gal80*^*ts*^*/UAS-dAtg1*^*RNAi*^); (3) *DE*^*ts*^ *> hid-dAtg1*^*RNAi*^ (*UAS-hid/+; UAS-GFP/+; DE-Gal4 tub-Gal80*^*ts*^*/UAS-dAtg1*^*RNAi*^); (4) *DE*^*ts*^ *> dAtg1*^*DN*^ (*UAS-GFP/+; DE-Gal4 tub-Gal80*^*ts*^*/UAS-dAtg1*^*DN*^); (5) *DE*^*ts*^ *> hid-dAtg1*^*DN*^ (*UAS-hid/+; UAS-GFP/+; DE-Gal4 tub-Gal80*^*ts*^*/UAS-dAtg1*^*DN*^) were raised at 18 °C. Expression of UAS-constructs (*GFP*, *hid*, *dAtg1*^*RNAi*^, *dAtg1*^*DN*^) was induced by a temporal temperature shift to 29 °C for 12 h. After a 72 h recovery period at 18 °C, late third instar eye discs were dissected and analyzed as indicated in the panels (Fig. 2). Full details of the *DE*^*ts*^ *> hid* assay have been described previously [52]. At least three independent experimental repeats were done for each genotype and the results were consistent. For statistical analysis shown in Fig. 2d, at least 10 eye discs from each of the following genotypes, *DE*^*ts*^ *> hid*; *DE*^*ts*^ *> dAtg1*^*RNAi*^; *DE*^*ts*^ *> hid-dAtg1*^*RNAi*^; *DE*^*ts*^ *> dAtg1*^*DN*^; and *DE*^*ts*^ *> hid-dAtg1*^*DN*^, were measured for their sizes of dorsal versus ventral half of discs using the “histogram” function in Adobe Photoshop CS6. For such measurement, location of the optic stalk at the center of the posterior edge of eye disc was used as a landmark to horizontally divide eye discs into dorsal versus ventral halves. The dorsal/ventral size ratio was then calculated for each genotype. The statistical significance was evaluated through a one-way ANOVA with Bonferroni multiple comparison test (at least *P* < 0.01). For the developing wing tissue (Fig. 5), *hh-Gal4 tub-Gal80*^*ts*^ (*hh*^*ts*^) was used to temporally control expression of UAS-constructs in the posterior compartment of wing discs.

### Mosaic analysis

For mosaic analysis with “undead” cell clones in larval discs (Fig. 4), the 3 L-MARCM assay was used [84]. Mid second instar (32–40 h post-hatching) larvae of the following genotypes were heat shocked for 1 h at 37 °C, raised at 25 °C, and analyzed at the late third instar larval stage. (1) Generation of *hid* and *p35* co-expressing “undead” clones: *hs-FLP tub-GAL4 UAS-GFP/UAS-hid; UAS-p35/+; tub-GAL80 FRT80B/FRT80B*. (2) Generation of *hid* and *p35* co-expressing *dronc* mutant clones: *hs-FLP tub-GAL4 UAS-GFP/UAS-hid; UAS-p35/+; tub-GAL80 FRT80B/dronc*^*I29*^*FRT80B*. (3) Generation of *hid* and *p35* co-expressing *dAtg1* mutant clones: *hs-FLP tub-GAL4 UAS-GFP/UAS-hid; UAS-p35/+; tub-GAL80 FRT80B/dAtg1*^*Δ3D*^*FRT80B*. (4) Generation of *dAtg1* mutant clones in *GMR-hid* eye discs: *ey-FLP/+; GMR-hid/+; dAtg1*^*Δ3D*^*FRT80B/ubi-GFP FRT80B*. The mosaic assay in starving fat body (Additional file 7: Figure S7) was done according to Neufeld [85]. *UAS-RNAi* lines targeting *dAtg1*, *dAtg3*, *dAtg8a*, and *dAtg8b* were crossed to *yw hs-FLP; r4-mCherry-Atg8a Act > CD2 > Gal4 UAS-GFPnls* [86] and incubated at 25 °C. Offspring were starved for 3 h on 20 % sucrose solution before dissection.

### Immunohistochemistry and quantification of cCasp3 labeling intensity

Imaginal discs were dissected from late third instar larvae and stained using standard protocols [87]. Antibodies to the following primary antigens were used: anti-cleaved Caspase-3 (Cell Signaling), β-GAL, ELAV, MMP1 (3B8D12 and 5H7B1 used as a 1:1 cocktail), and Wg (all DHSB). dATG1 antibodies were kindly provided by Jun Hee Lee [71]. Secondary antibodies were donkey Fab fragments conjugated to FITC, Cy3 or Cy5 from Jackson ImmunoResearch. For the dATG1 labeling, HRP-labeled secondary antibodies were used and amplified with Tyramide Signal Amplification (TSA, PerkinElmer). Fluorescent images were taken with a Zeiss confocal microscope. Adult fly images were taken using a Zeiss stereomicroscope equipped with an AxioCam ICC1 camera.

For quantification of cCasp3 labeling intensity in eye or wing discs (Fig. 3j, k and Additional file 3: Figure S3C), the average cCasp3 signal intensities in certain disc areas were acquired through Adobe Photoshop CS6 and normalized to the corresponding background level of cCasp3 labeling in the same disc. The background cCasp3 labeling intensity was obtained from the antenna discs for measurement in eye discs (Fig. 3j), the notum regions for measurement in wing discs (Fig. 3k), and the non-clonal areas for the Additional file 3: Figure S3C. At least five representative discs of each genotype were used for such quantification. The statistical significance was evaluated through either a one-way ANOVA with Bonferroni multiple comparison test (at least *P* < 0.01, Fig. 3j, k) or a two-tailed, unpaired Student’s *t* test (Additional file 3: Figure S3C).

### In situ hybridization

For in situ hybridization to detect *dAtg1* transcripts, *Drosophila* cDNA clone LD18893 (Berkeley *Drosophila* Genome Project expressed sequence tags, *Drosophila* Genomic Resource Center) was used as a template to generate digoxigenin-labeled sense and antisense RNA probes (Roche). Labeled probes were detected with a TSA Cy3 kit (PerkinElmer) as previously described [88].

### Quantitative real-time PCR (qPCR)

Total RNA was isolated from 100 eye discs collected from either the control *ey-GAL4* or *ey-GAL4 UAS-Atg1RNAi* (*ey > dAtg1*^*RNAi*^) third instar larvae using the TRIzol Reagent (Thermo Fisher Scientific). cDNA was then generated from 1 μg of total RNA with the GoScript™ Reverse Transcription System (Promega). This is followed by the real-time PCR using the SensiFAST SYBR Hi-Rox kit (BIOLINE) with a ABI Prism7000 system (Life technologies). *dAtg1* mRNA levels were normalized to the reference gene *ribosomal protein L32* (*RPL32*) by using the ΔΔCt analysis. Three independent biological repeats were analyzed. The following primers suggested by the FlyPrimerBank [89] were used: *dAtg1* Fw, CGTCAGCCTGGTCATGGAGTA; *dAtg1* Rv, TAACGGTATCCTCGCTGAG; *RPL32* Fw, AGCATACAGGCCCAAGATCG; *RPL32* Rv, TGTTGTCGATACCCTTGGGC.

## Additional files

Additional file 1: Figure S1. Specificity of dATG1 antibodies and *dAtg1* ^*RNAi*^. (**A**) *dAtg1* transcript levels were determined by qPCR from total RNA extracted from eye discs without (control) or with expression of *dAtg1*RNAi driven by *ey > GAL4. dAtg1* RNAi suppresses *dAtg1* transcript levels to less than 30 %. Error bars represent SD of three biological repeats. (**B, B’**) A *hh > dAtg1* ^*RNAi*^ wing disc labeled with dATG1 antibodies (red in B, grey in B’). Expression of *dAtg1* is strongly reduced in the posterior compartment (GFP^+^) where *dAtg1* ^*RNAi*^ is expressed. (**C, C’**) A *GMR > dAtg1* eye disc labeled with dATG1 antibodies (red in C, grey in C’). ATG1 antibodies specifically recognize dATG1 proteins expressed in the *GMR* domain (GFP^+^). (TIF 1956 kb) Additional file 2: Figure S2. Expression of *dAtg1* enhances caspase activity and apoptosis. Late third instar larval eye discs labeled with the cleaved Caspase-3 antibodies (cCasp3, green in A, B, grey in A’, B’, blue in C, and grey in C’), anterior is to the left. (**A–B’**) Compared to *ey > hid-p35* discs (A, A’), cCasp3 labeling indicating activity of Dronc is not affected by expression of *dAtg1* which enhances *ey > hid-p35*-induced overgrowth phenotype (B, B’). (**C–C”’**) Expression of *dAtg1* under control of *DE-Gal4* and *tub-Gal80ts* (*DEts*) and indicated by GFP. Expression of *dAtg1* by a temperature shift (ts) to 29 °C for 48 h induces apoptosis as indicated by cCasp3 labeling (C’, arrow) and developmental defects in the eye disc indicated by the affected pattern of ELAV labeling (C”, arrow). (TIF 4775 kb) Additional file 3: Figure S3. Loss of *dAtg1* does not suppress apoptosis. (**A–B’**) Mosaic late third instar wing discs with *hid*-*p35*-expressing clones positively marked by GFP. Simultaneous expression of *hid* and *p35* in clones induces strong cCasp3 labeling (A, A’, arrows). Similar cCasp3 labeling persists in *dAtg1* mutant clones (B, B’, arrows). (**C**) Quantification of cCasp3 labeling intensity in *hid-p35*-expressing clones and *hid-p35*-expressing *dAtg1* mutant clones (mean ± SE). No significant difference of cCasp3 labeling was observed. (**D–D’**) A representative late third instar *GMR-hid* eye disc with *dAtg1* mutant clones negatively marked by GFP (highlighted by yellow dotted lines). The wave of apoptosis (arrow) induced by *GMR-hid* persists in *dAtg1* mutant clones. (**E, F**) Representative adult eyes of the indicated genotypes. *GMR-hid*-induced eye ablation phenotype (**E**) is not altered by RNAi knockdown of *dAtg1* (**F**). (TIF 6416 kb) Additional file 4: Figure S4. Expression of *dAtg1* is not sufficient to induce growth signals for AiP. Late third instar eye discs labeled with *wg-lacZ* (red in B, C and grey in A, B’, C’), *dpp-lacZ* (red in E, F and grey in D, E’,F’) or *kekkon-lacZ* (*kek-lacZ*, red in H, I and grey in G, H’, I’). Anterior is to the left. *DE-Gal4 tub-Gal80* ^*ts*^ (*DE* ^*ts*^) was used to control expression of *UAS*-transgenes at 29 °C for 12 h in the dorsal portion of eye discs, followed by 24 h of recovery at 18 °C (TS12hR24h). Compared to control discs (A, D, G), temporal expression of *hid* leads to apoptosis, indicated by the cCasp3 labeling (green in B), and ectopic induction of *wg-lacZ* (B’, arrow), *dpp-lacZ* (E’, arrow) and *kek-lacZ* (H’, arrow) which are markers of the growth signaling pathways mediating AiP. In contrast, expression of *dAtg1* under the same conditions (TS12hR24h) does not activate ectopic *wg*, *dpp* or *kek* (compare C’, F’, I’ to B’, E’, H’) although a low level of apoptosis is induced (cCasp3-labeling, green in C). (TIF 7173 kb) Additional file 5: Figure S5. Specificity of in situ probes to detect *dAtg1* transcripts. In situ hybridization of late third instar larval eye discs with DIG-labeled probes detected with Tyramide Signal Amplification. (**A**) Endogenous *dAtg1* is expressed at low level in wildtype eye discs. (**B, C**) Labeling of *GMR > dAtg1* discs using sense probes (B) and anti-sense *dAtg1* probes (C). The *dAtg1* antisense probes recognize high levels of *dAtg1* transcripts driven by *GMR-Gal4* (C, the *GMR* domain expressing *dAtg1* is highlighted). (TIF 1140 kb) Additional file 6: Figure S6. (**A–C”**) Autophagic flux reporter expression in *ey > hid-p35* eye discs. Late third instar larval eye discs expressing the autophagic flux reporter *GFP-mCherry-dAtg8*a under control of the *dAtg8* promoter [76]. The yellow dotted lines indicate the anterior portions of the eye discs which expresses *ey-Gal4*. Note the overgrowth of the anterior eye disc portion in *ey* > *hid*-*p35* imaginal discs (B–B”). Expression of GFP and mCherry is low in the control *ey > p35* discs (A–A”). In contrast, the numbers of GFP and mCherry positive particles are strongly increased in the overgrown *ey-Gal4* expressing area of *ey > hid-p35* discs (B–B”). Although the overgrowth of *ey* > *hid*-*p35* eye discs is strongly suppressed by *dAtg1* RNAi, the GFP and mCherry signals are not significantly reduced (C–C”). (TIF 7456 kb) Additional file 7: Figure S7. Functional tests of the RNAi lines targeting *dAtg1*, *dAg3*, *dAtg8a*, and *dAtg8b*. (A–E) Starvation assay of fat bodies from third instar larvae. Formation of autophagosomes was visualized by *mCherry-Atg8* (red in A–E; grey in A’–E’). Cells expressing RNAi constructs are labeled by GFP and outlined by yellow dotted lines. (A) Wildtype fat body displaying *mCherry-Atg8* puncta both in clone cells and surrounding cells. (B–E) Cells expressing *dAtg1*, *dAtg3*, *dAtg8a*, and *dAtg8b* RNAi (*GFP* ^+^) fail to form *mCherry-Atg8* marked autophagosomes. The loss of *mCherry-Atg8* signals by *dAtg8a* and *dAtg8b* RNAi in (D) and (E) also demonstrates that these RNAi lines target *mCherry-Atg8* transcripts. (**F–J**) Adult eyes expressing *eyeful* and indicated RNAi transgenes. As previously reported [77], loss of autophagy strongly enhances the *eyeful* phenotype. The functionality of *dAtg1*, *dAtg3*, *dAtg8a*, and *dAtg8b* RNAi transgenes is confirmed by enhancement of the *eyeful* phenotype. (TIF 8420 kb)
