# Supplementary figures and images for: Autophagy-independent function of Atg1 for apoptosis-induced compensatory proliferation
Source: BMC Biol. 2016 Aug 19;14:70. doi: 10.1186/s12915-016-0293-y (PMC4992243; doi:10.1186/s12915-016-0293-y)

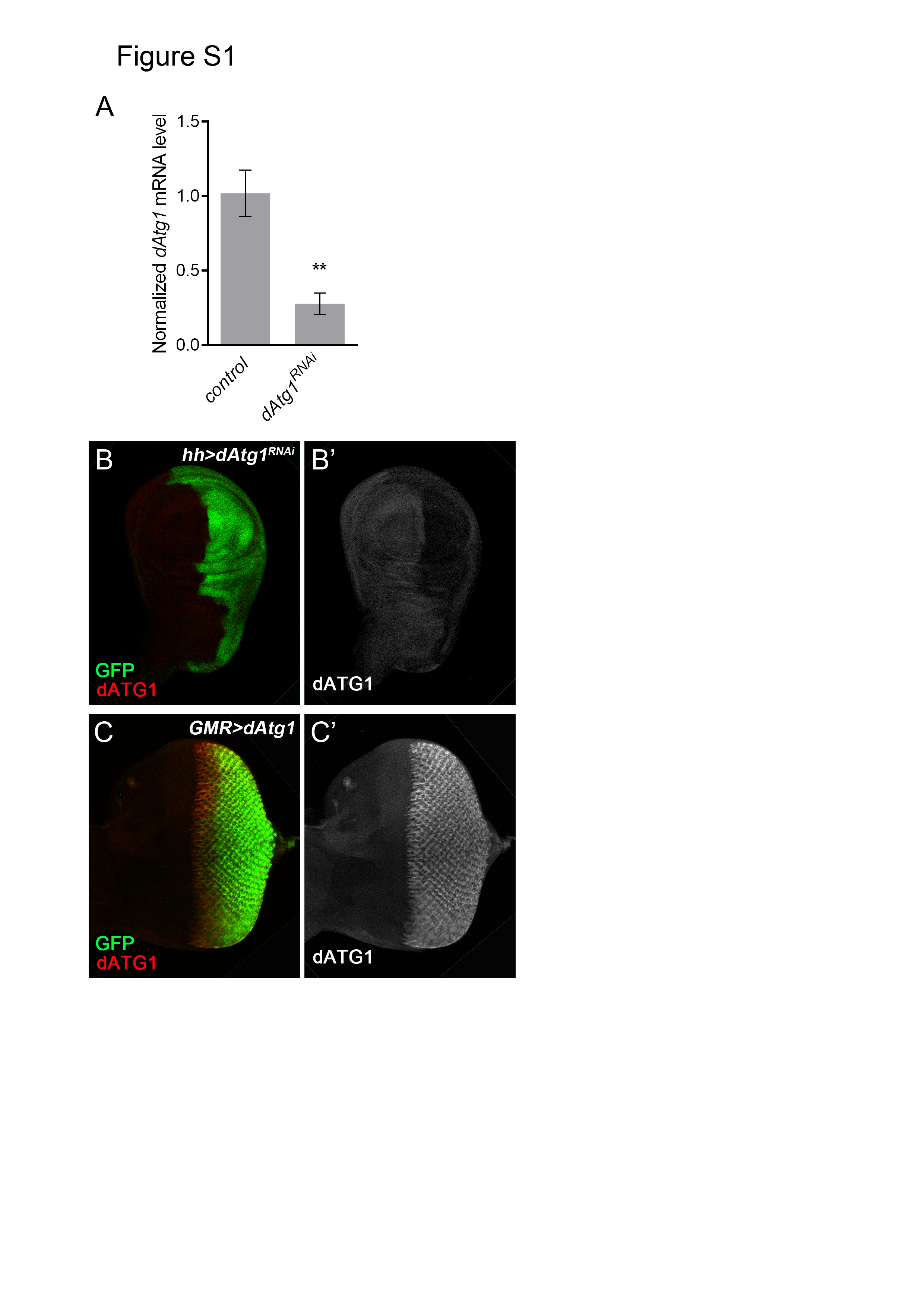

Supplement: Additional file 1: Figure S1. — Specificity of dATG1 antibodies and dAtg1 RNAi. (A) dAtg1 transcript levels were determined by qPCR from total RNA extracted from eye discs without (control) or with expression of dAtg1RNAi driven by ey > GAL4. dAtg1 RNAi suppresses dAtg1 transcript levels to less than 30 %. Error bars represent SD of three biological repeats. (B, B’) A hh > dAtg1 RNAi wing disc labeled with dATG1 antibodies (red in B, grey in B’). Expression of dAtg1 is strongly reduced in the posterior compartment (GFP+) where dAtg1 RNAi is expressed. (C, C’) A GMR > dAtg1 eye disc labeled with dATG1 antibodies (red in C, grey in C’). ATG1 antibodies specifically recognize dATG1 proteins expressed in the GMR domain (GFP+). (TIF 1956 kb) [file 12915_2016_293_MOESM1_ESM.tif]

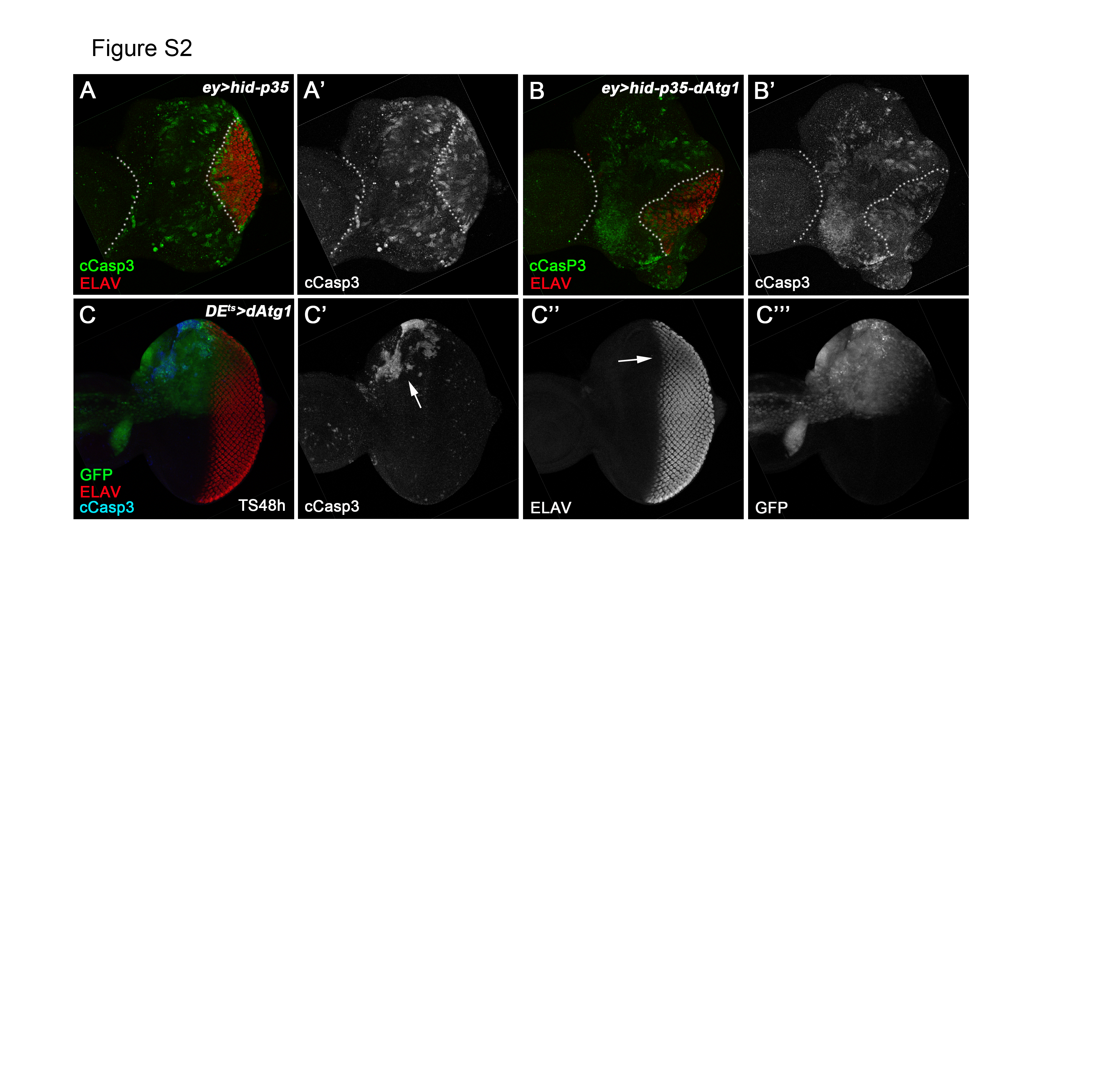

Supplement: Additional file 2: Figure S2. — Expression of dAtg1 enhances caspase activity and apoptosis. Late third instar larval eye discs labeled with the cleaved Caspase-3 antibodies (cCasp3, green in A, B, grey in A’, B’, blue in C, and grey in C’), anterior is to the left. (A–B’) Compared to ey > hid-p35 discs (A, A’), cCasp3 labeling indicating activity of Dronc is not affected by expression of dAtg1 which enhances ey > hid-p35-induced overgrowth phenotype (B, B’). (C–C”’) Expression of dAtg1 under control of DE-Gal4 and tub-Gal80ts (DEts) and indicated by GFP. Expression of dAtg1 by a temperature shift (ts) to 29 °C for 48 h induces apoptosis as indicated by cCasp3 labeling (C’, arrow) and developmental defects in the eye disc indicated by the affected pattern of ELAV labeling (C”, arrow). (TIF 4775 kb) [file 12915_2016_293_MOESM2_ESM.tif]

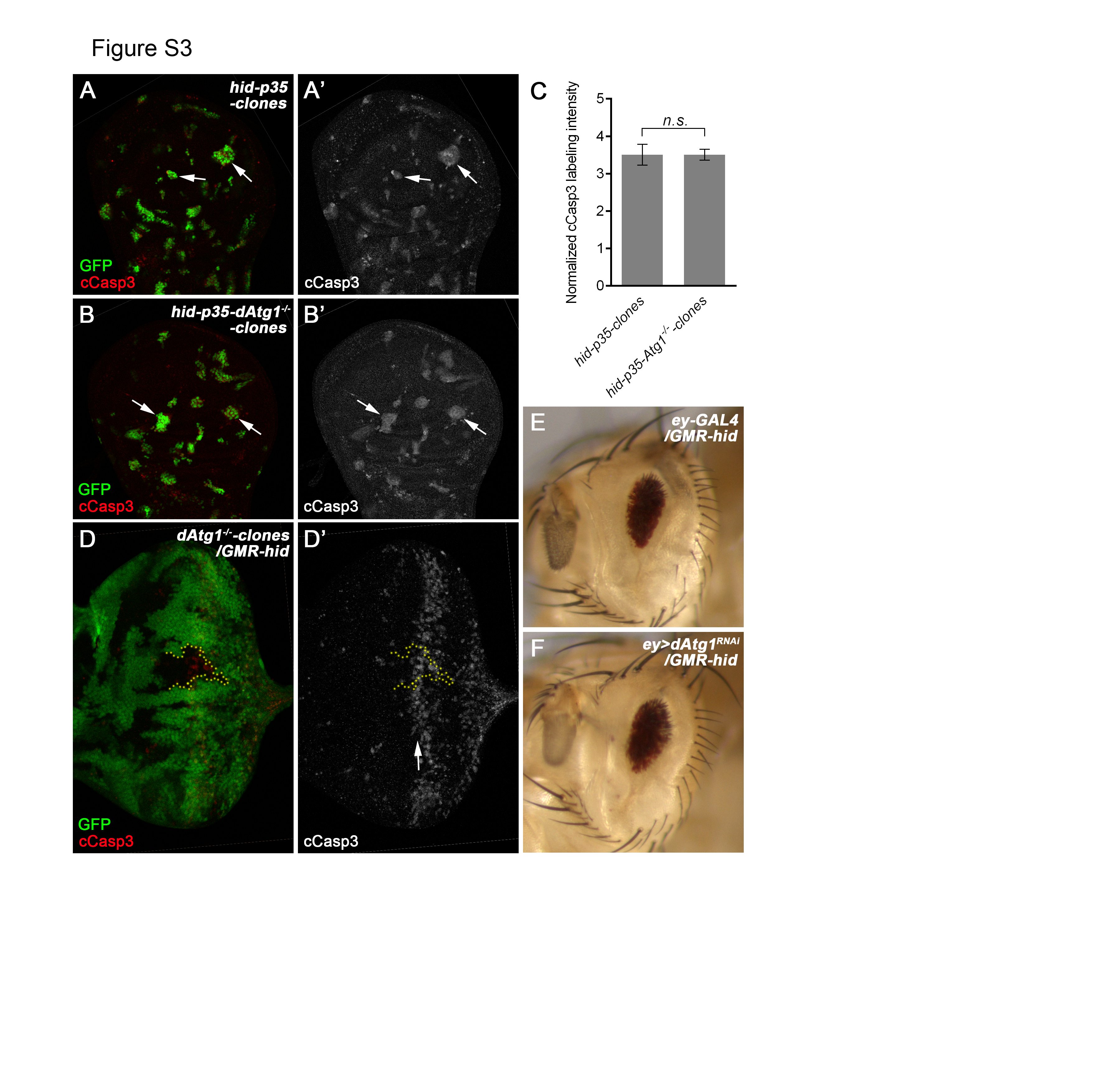

Supplement: Additional file 3: Figure S3. — Loss of dAtg1 does not suppress apoptosis. (A–B’) Mosaic late third instar wing discs with hid-p35-expressing clones positively marked by GFP. Simultaneous expression of hid and p35 in clones induces strong cCasp3 labeling (A, A’, arrows). Similar cCasp3 labeling persists in dAtg1 mutant clones (B, B’, arrows). (C) Quantification of cCasp3 labeling intensity in hid-p35-expressing clones and hid-p35-expressing dAtg1 mutant clones (mean ± SE). No significant difference of cCasp3 labeling was observed. (D–D’) A representative late third instar GMR-hid eye disc with dAtg1 mutant clones negatively marked by GFP (highlighted by yellow dotted lines). The wave of apoptosis (arrow) induced by GMR-hid persists in dAtg1 mutant clones. (E, F) Representative adult eyes of the indicated genotypes. GMR-hid-induced eye ablation phenotype (E) is not altered by RNAi knockdown of dAtg1 (F). (TIF 6416 kb) [file 12915_2016_293_MOESM3_ESM.tif]

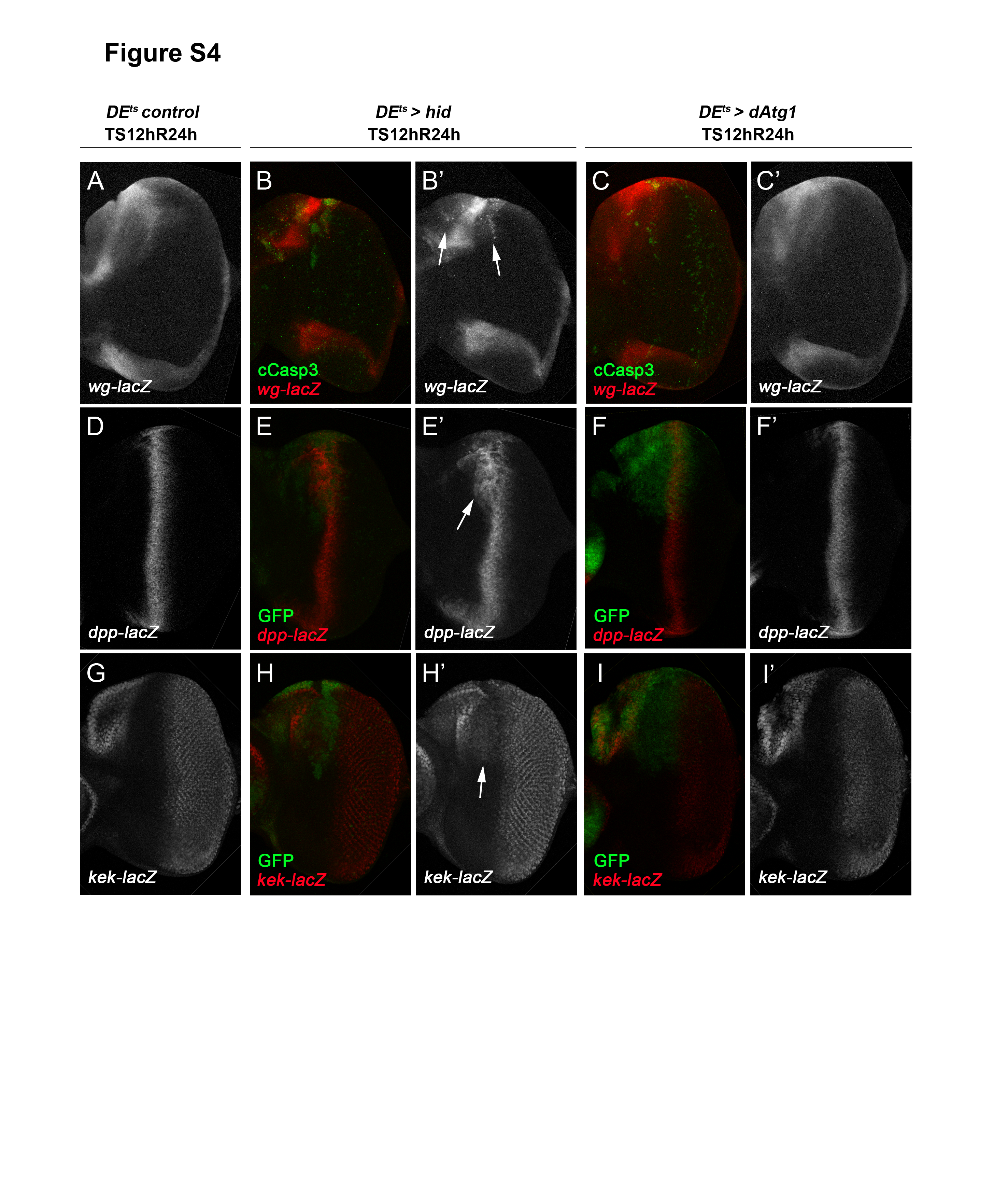

Supplement: Additional file 4: Figure S4. — Expression of dAtg1 is not sufficient to induce growth signals for AiP. Late third instar eye discs labeled with wg-lacZ (red in B, C and grey in A, B’, C’), dpp-lacZ (red in E, F and grey in D, E’,F’) or kekkon-lacZ (kek-lacZ, red in H, I and grey in G, H’, I’). Anterior is to the left. DE-Gal4 tub-Gal80 ts (DE ts) was used to control expression of UAS-transgenes at 29 °C for 12 h in the dorsal portion of eye discs, followed by 24 h of recovery at 18 °C (TS12hR24h). Compared to control discs (A, D, G), temporal expression of hid leads to apoptosis, indicated by the cCasp3 labeling (green in B), and ectopic induction of wg-lacZ (B’, arrow), dpp-lacZ (E’, arrow) and kek-lacZ (H’, arrow) which are markers of the growth signaling pathways mediating AiP. In contrast, expression of dAtg1 under the same conditions (TS12hR24h) does not activate ectopic wg, dpp or kek (compare C’, F’, I’ to B’, E’, H’) although a low level of apoptosis is induced (cCasp3-labeling, green in C). (TIF 7173 kb) [file 12915_2016_293_MOESM4_ESM.tif]

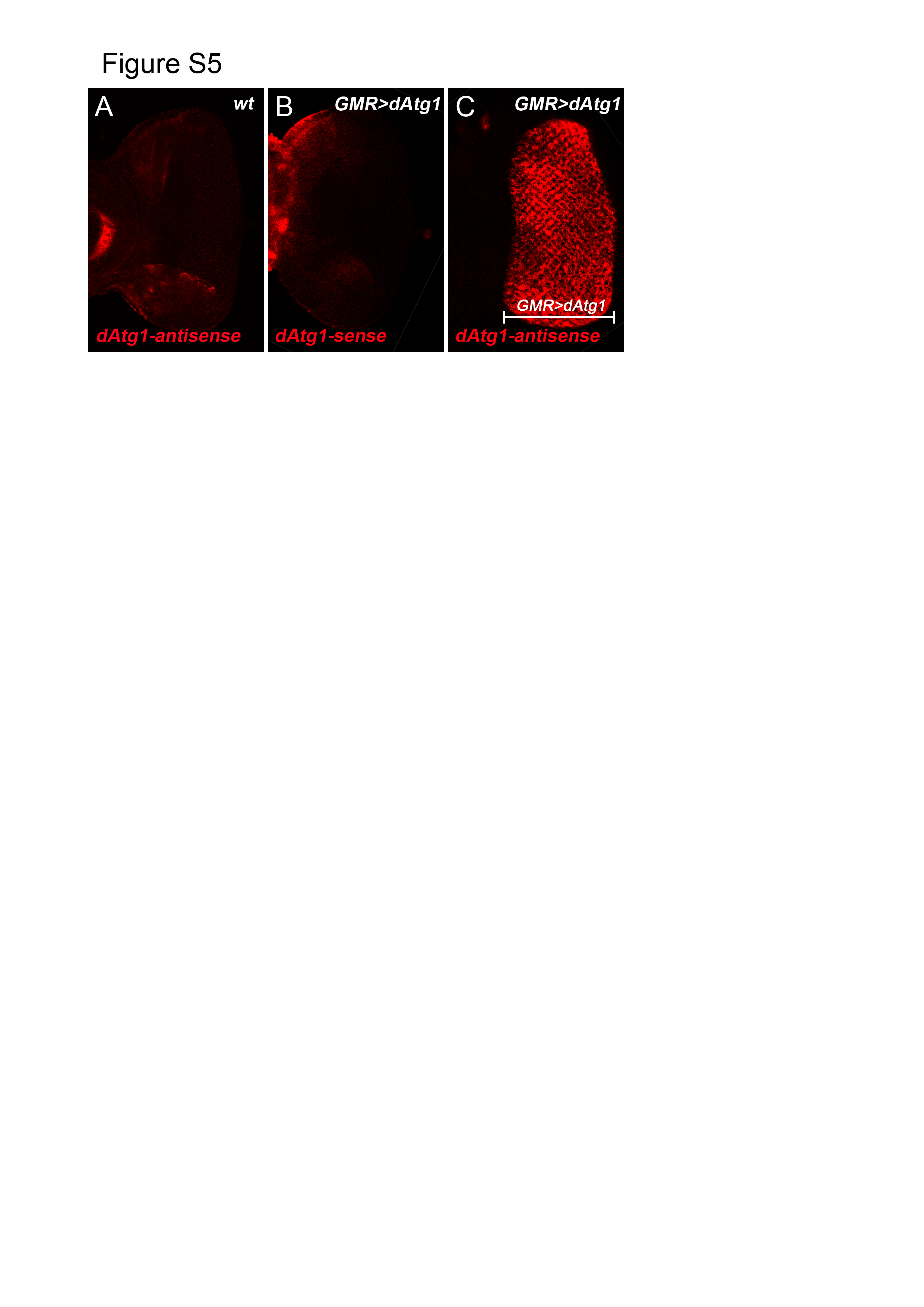

Supplement: Additional file 5: Figure S5. — Specificity of in situ probes to detect dAtg1 transcripts. In situ hybridization of late third instar larval eye discs with DIG-labeled probes detected with Tyramide Signal Amplification. (A) Endogenous dAtg1 is expressed at low level in wildtype eye discs. (B, C) Labeling of GMR > dAtg1 discs using sense probes (B) and anti-sense dAtg1 probes (C). The dAtg1 antisense probes recognize high levels of dAtg1 transcripts driven by GMR-Gal4 (C, the GMR domain expressing dAtg1 is highlighted). (TIF 1140 kb) [file 12915_2016_293_MOESM5_ESM.tif]

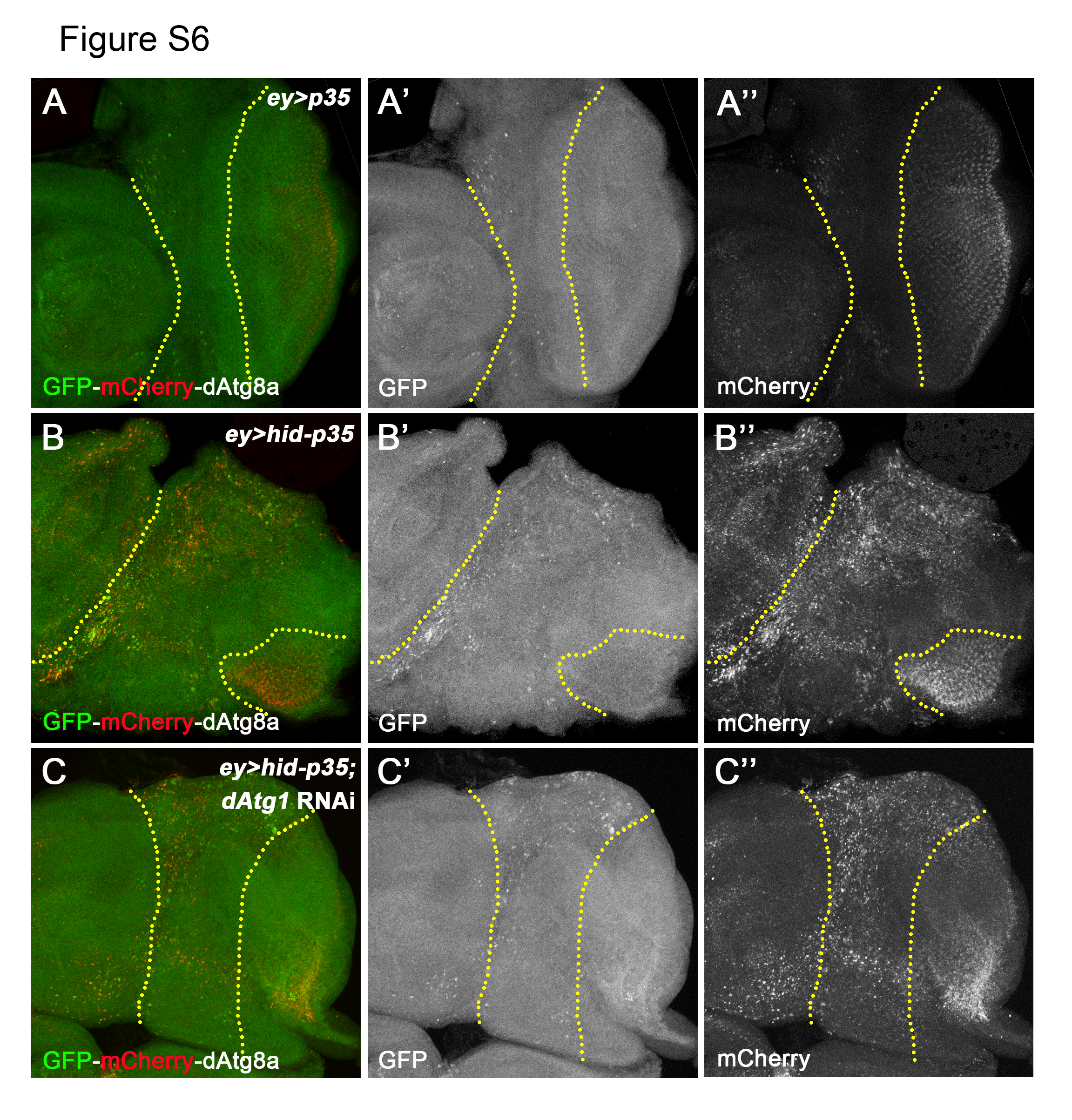

Supplement: Additional file 6: Figure S6. — (A–C”) Autophagic flux reporter expression in ey > hid-p35 eye discs. Late third instar larval eye discs expressing the autophagic flux reporter GFP-mCherry-dAtg8a under control of the dAtg8 promoter [76]. The yellow dotted lines indicate the anterior portions of the eye discs which expresses ey-Gal4. Note the overgrowth of the anterior eye disc portion in ey > hid-p35 imaginal discs (B–B”). Expression of GFP and mCherry is low in the control ey > p35 discs (A–A”). In contrast, the numbers of GFP and mCherry positive particles are strongly increased in the overgrown ey-Gal4 expressing area of ey > hid-p35 discs (B–B”). Although the overgrowth of ey > hid-p35 eye discs is strongly suppressed by dAtg1 RNAi, the GFP and mCherry signals are not significantly reduced (C–C”). (TIF 7456 kb) [file 12915_2016_293_MOESM6_ESM.tif]

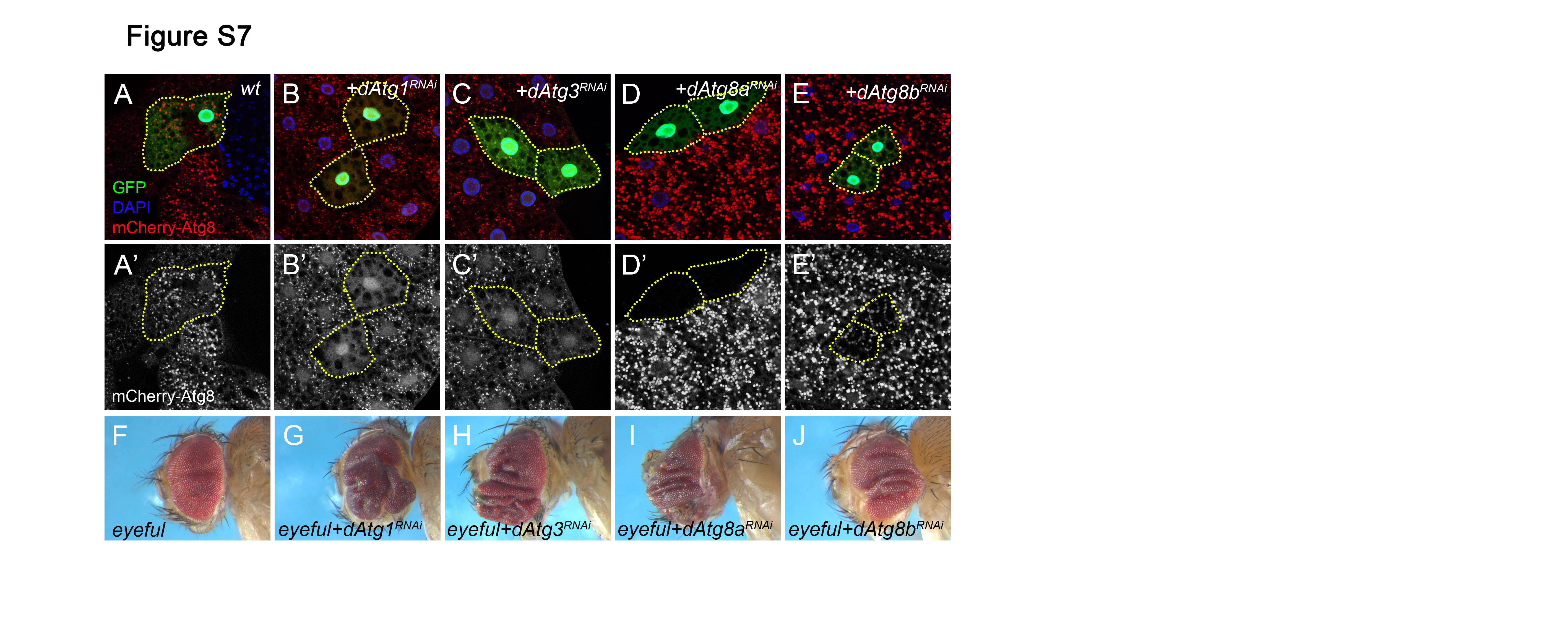

Supplement: Additional file 7: Figure S7. — Functional tests of the RNAi lines targeting dAtg1, dAg3, dAtg8a, and dAtg8b. (A–E) Starvation assay of fat bodies from third instar larvae. Formation of autophagosomes was visualized by mCherry-Atg8 (red in A–E; grey in A’–E’). Cells expressing RNAi constructs are labeled by GFP and outlined by yellow dotted lines. (A) Wildtype fat body displaying mCherry-Atg8 puncta both in clone cells and surrounding cells. (B–E) Cells expressing dAtg1, dAtg3, dAtg8a, and dAtg8b RNAi (GFP +) fail to form mCherry-Atg8 marked autophagosomes. The loss of mCherry-Atg8 signals by dAtg8a and dAtg8b RNAi in (D) and (E) also demonstrates that these RNAi lines target mCherry-Atg8 transcripts. (F–J) Adult eyes expressing eyeful and indicated RNAi transgenes. As previously reported [77], loss of autophagy strongly enhances the eyeful phenotype. The functionality of dAtg1, dAtg3, dAtg8a, and dAtg8b RNAi transgenes is confirmed by enhancement of the eyeful phenotype. (TIF 8420 kb) [file 12915_2016_293_MOESM7_ESM.tif]
